# Increased E2F2 predicts poor prognosis in patients with HCC based on TCGA data

**DOI:** 10.1186/s12885-020-07529-2

**Published:** 2020-10-28

**Authors:** Zhili Zeng, Zebiao Cao, Ying Tang

**Affiliations:** 1 Guangzhou University of Chinese Medicine, Guangzhou, 510405 Guangdong China; 2 Lingnan Medical Research Center of Guangzhou University of Chinese Medicine, no.12, Airport Road, Sanyuanli Street, Baiyun District, Guangzhou, 510405 Guangdong China

**Keywords:** Hepatocellular carcinoma, E2F2, Prognosis

## Abstract

**Background:**

The E2F family of transcription factor 2 (E2F2) plays an important role in the development and progression of various tumors, but its association with hepatocellular carcinoma (HCC) remains unknown. Our study aimed to investigate the role and clinical significance of E2F2 in HCC.

**Methods:**

HCC raw data were extracted from The Cancer Genome Atlas (TCGA). Wilcoxon signed-rank test, Kruskal-Wallis test and logistic regression were applied to analyze the relationship between the expression of E2F2 and clinicopathologic characteristics. Cox regression and Kaplan-Meier were employed to evaluate the correlation between clinicopathologic features and survival. The biological function of E2F2 was annotated by Gene Set Enrichment Analysis (GSEA).

**Results:**

The expression of E2F2 was increased in HCC samples. The expression of elevated E2F2 in HCC samples was prominently correlated with histologic grade (OR = 2.62 for G3–4 vs. G1–2, *p* = 1.80E-05), clinical stage (OR = 1.74 for III-IV vs. I-II, *p* = 0.03), T (OR = 1.64 for T3–4 vs.T1–2, *p* = 0.04), tumor status (OR = 1.88 for with tumor vs. tumor free, *p* = 3.79E-03), plasma alpha fetoprotein (AFP) value (OR = 3.18 for AFP ≥ 400 vs AFP<20, *p* = 2.16E-04; OR = 2.50 for 20 ≤ AFP<400 vs AFP<20, *p* = 2.56E-03). Increased E2F2 had an unfavorable OS (*p* = 7.468e− 05), PFI (*p* = 3.183e− 05), DFI (*p* = 0.001), DSS (*p* = 4.172e− 05). Elevated E2F2 was independently bound up with OS (*p* = 0.004, hazard ratio [HR] = 2.4 (95% CI [1.3–4.2])), DFI (*P* = 0.029, hazard ratio [HR] = 2.0 (95% CI [1.1–3.7])) and PFI (*P* = 0.005, hazard ratio [HR] = 2.2 (95% CI [1.3–3.9])). GSEA disclosed that cell circle, RNA degradation, pyrimidine metabolism, base excision repair, aminoacyl tRNA biosynthesis, DNA replication, p53 signaling pathway, nucleotide excision repair, ubiquitin-mediated proteolysis, citrate cycle TCA cycle were notably enriched in E2F2 high expression phenotype.

**Conclusions:**

Elevated E2F2 can be a promising independent prognostic biomarker and therapeutic target for HCC. Additionally, cell cycle, pyrimidine metabolism, DNA replication, p53 signaling pathway, ubiquitin-mediated proteolysis, the citrate cycle TCA cycle may be the key pathway by which E2F2 participates in the initial and progression of HCC.

## Background

Primary liver cancer is a common malignant tumor with high morbidity and mortality. It is the fourth leading cause of cancer mortality and the sixth leading cause of cancer incidence in the world. Hepatocellular carcinoma (HCC) is the main type of primary liver cancer, in this paper, we chiefly focus on HCC in this study. The world health organization predicts that one million patients will die of HCC by 2030 [1]. Surgical resection and liver transplantation are effective means to cure early liver cancer. However, there are still many patients with postoperative recurrence and metastasis. Even with successful surgical resection or liver transplantation, the 5-year survival rate of patients is only 36–70% and 60–70%, respectively [2]. Therefore, the effective prediction of prognosis is of great significance to improve the 5-year survival rate of patients. On the one hand, prediction of prognosis is helpful to encourage patients with poor prognosis to strengthen monitoring of abnormal indicators after treatment, once the abnormality is found, they can be treated as soon as possible; On the other hand, it can help doctors develop more effective treatment plans and determine whether appropriate adjuvant therapy is needed to prevent recurrence and metastasis, prognosis assessment is a key step in the proper management of HCC patients [3]. At present, alpha fetoprotein (AFP) and ultrasound can only be used as the indicators of HCC screening, and so far there has been yet no strong biomarker for early prediction of patient prognosis.

Previous studies have reported that the E2F family of transcription factor 2 (E2F2), as an important member of the E2F family, has important correlations with various cancer types, and has different expressions and functions in different tumors. It has been discovered that E2F2 is prominently upregulated in NSCLC, and can serve as a therapeutic target to prevent the proliferation and invasion of NSCLC [4]. Quan Zhou et al. reported that overexpressed E2F2 is closely related to poor post progression survival in ovarian cancer patients, and can be used for targeted treatment and prognosis prediction [5]. Similar results can be found in glioma [6], osteosarcoma [7], gastric cancer [8] and melanoma [9], it’s not surprising that E2F2 is regarded as an oncogene. However, E2F2 is a suppressor gene in clear cell renal cell carcinoma [10] and T-cell lymphoma [11]. What is the role of E2F2 in the HCC? Few papers have reported the relationship between E2F2 and HCC. Up to now, there has been no report on E2F2 in predicting the prognosis of HCC. Our present study aimed to explore the correlation between E2F2 and HCC, and to evaluate the prognostic value of E2F2 in HCC, as well as the possible mechanism by which E2F2 affects the prognosis of HCC.

## Methods

### RNA-sequencing genes expression profiles and clinical information

The gene expression data and corresponding clinical information were extracted from The Cancer Genome Atlas (TCGA) database (https://portal.gdc.cancer.gov/repository). The inclusion criteria are (1) primary hepatocellular carcinoma; (2) complete RNA-seq data. The exclusion criterion is that (1) there is not enough data in the sample for analysis, such as insufficient survival information; (2) the clinicopathological feature information is ambiguous. A total of 374 HCC cases and 50 normal cases were included in the present study, and the workflow type was HTSeq-FPKM. The clinical characteristics of patients involving age, gender, Body Mass Index (BMI), family history of cancer, grade, stage, topography (T), lymph node (N), metastasis (M), residual tumor, tumor status, vascular invasion, Child-Pugh, AFP, new tumor event, history of alcohol consumption, postoperative ablation embolization and were downloaded. Some patients have incomplete clinicopathological information, so these patients are included in the analysis of clinical information they have and excluded from the analysis of clinicopathological characteristics they lack. Therefore, the total number of some variables in Table 1 is not 374. Survival analysis such as overall survival (OS) was measured from the time of study enrollment to the day of death due to any cause or last follow-up. Disease-specific Survival (DSS) was recorded as the time between the day of diagnosis or initiation of treatment for HCC and the day of death due to HCC. The disease-free interval (DFI) was defined as the time from the day of curative surgery of intrahepatic lesion to the day of the first detection of recurrence and metastasis. Progression-free interval (PFI) was defined as the time between the date of diagnosis of HCC and the date of the first detection of progression or loss of follow-up. The median follow-up time for OS and DSS was 37.4 months (range 0–192 months); The median follow-up time for DFI and PFI was 37.4 months (range 0–192 months).

**Table 1:** HCC patient characteristics based on TCGA

Clinical characteristics		Total (424)	%
Age (years)	>40	342	91.0
	≤40	34	9.0
Gender	male	255	67.6
	female	122	32.4
BMI	≥25	161	47.2
	<25	180	52.8
Family history of cancer	Yes	114	35.0
	No	212	65.0
Histologic grade	G1-G2	235	63.1
	G3-G4	137	36.8
Clinical stage	I-II	262	74.2
	III-IV	91	25.8
T	T1-T2	280	74.9
	T3-T4	94	25.1
N	N0	257	98.5
	N1	4	1.5
M	M0	272	98.6
	M1	4	1.4
Residual tumor	R0	330	94.8
	R1	18	5.2
Tumor status	tumor free	236	67.6
	with tumor	113	32.4
Vascular invasion	Yes	111	34.6
	No	210	65.4
Child-pugh	A	223	91.0
	B-C	22	9.0
AFP	AFP<20	152	43.6
	20<AFP < 400	67	19.2
	AFP ≥ 400	130	37.2
New tumor event	Yes	98	36.0
	No	174	64.0
History of alcohol consumption	Yes	118	33.0
	No	240	67.0
Postoperative ablation embolization	Yes	28	7.9
	No	325	92.1
Radiation therapy	Yes	8	2.3
	No	344	97.7

T = topography distribution; N = lymph node metastasis; M = distant metastasis; *AFP* Alpha fetal protein

Furthermore, in order to validate the expression level of E2F2 mRNA in patients with HCC, we downloaded the raw gene profiles of GSE124535 and GSE54236 from the Gene Expression Omnibus (GEO) database.

The protein expression level of E2F2 was verified by the Human Protein Atlas (HPA) database (http://www.proteinatlas.org/) [12]. HPA aims to map the biology of all human proteins in cells, tissues and organs by integrating various omics techniques. Immunohistochemical (IHC) images were downloaded from the HPA database. The mean integrated optical density (IOD) value of IHC images was measured by Image-Pro Plus software (version 6.0; Media Cybernetics, Inc.). The higher the total IOD value, the greater the expression of E2F2. Using a non-paired T test in the GraphPad Prism® version 8.0 software to analyze the data of IHC. *P* < 0.05 was considered statistically significant.

### Enrichment analysis of GSEA

In the present study, the significant survival difference between the high and low level of E2F2 groups was illustrated through GSEA. Gene set enrichment analysis (GSEA) is a computational method, which can determine whether an apriori defined set of genes shows statistically significant, concordant differences between two biological states [13]. The number of gene set permutations were 1000 times for each analysis. The expression level of E2F2 was used as a phenotype label. The significantly enriched pathways were analyzed based on a normal *p*-value < 0.05 and false discovery rate (FDR) q-val < 0.05.

### Establishment of protein-protein interaction (PPI) network

To establish the interaction between E2F2 and its upstream and downstream targets in HCC, a E2F2-associated PPI network was constructed based on the Search Tool for the Retrieval of Interacting Genes/Proteins database (STRING) (https://string-db.org.uk/) [14] with a minimum required interaction score of > 0.9. Then Interactions were analyzed and visualized by Cytoscape v3.7.1 [15].

### Statistical analysis

Comparisons of the expression of E2F2 between HCC and normal groups were conducted using Wilcoxon rank sum tests via the “limma” and “beeswarm” packages of R software; and adjacent normal groups with Wilcoxon signed-rank tests. The correlations between the expression of E2F2 and clinicopathologic features were performed with Wilcoxon signed-rank test or Kruskal-Wallis test and logistic regression. The relationship between E2F2 expression and survival along with other clinicopathological features was performed with Cox regression analysis (“survival” package of R software was used in univariate Cox regression analysis, while “survival” and “survminer” packages of R software was used in multivariate Cox regression analysis) and the Kaplan–Meier method. In the Cox regression analysis, *P* < 0.05 indicated statistical significance. All statistical analyses were performed using R (version 3.6.1, 2019-07-05, R Foundation, Vienna, Austria).

## Results

### Clinical characteristics of patients

The data (shown in Table 1) were extracted from TCGA in December 2019 and included 374 primary HCC cases with both clinical and gene expression data. Clinical characteristics of HCC patients involving age, gender, BMI, family history of cancer, histologic grade, clinical stage, topography (T), lymph node (N), metastasis (M), residual tumor, tumor status, vascular invasion, Child-Pugh, AFP, new tumor event,. These patients included 122 female patients and 255 male patients,and most of them (91.0%, *n* = 342) were over 40 years old. The tumor status included 236 (67.6%) tumor free and 113 (32.4%) with tumor. In the study cohort, 161 of 341 (47.2%) patients were overweight and had a BMI of more than 25,114 of 326 (35.0%) patients had HCC family history, 118 of 358 (33.0%) had a history of alcohol consumption. As for tumor grade, there were 235 (63.1%) in G1 and G2, 137 (36.8%) were in G3 and G4. The cancer stage included 262 (74.2%) stage I and stage II, 91 (25.8%) stage III and stage IV. The topography of patients included 280 (74.9%) T1 and T2, 94 (25.1%) T3 and T4. A total of 4 of 261 (1.5%) cases had lymph node metastasis and 4 of 276 (1.4%) cases had distant metastases, 111 of 321 (34.6%) cases had a vascular invasion. 223(91.0%) patients were found in Child-Pugh A, 22 (9.0%) patients were found in Child-Pugh B and C. The distribution of AFP values included 43.6% (*n* = 152) AFP<20, 19.2% (*n* = 67) 20 ≤ AFP < 400, 37.2% (*n* = 130) AFP ≥ 400. A total of 8 of 352 (2.3%) patients underwent radiation therapy. After the operation, 28 of 353 (7.9%) patients had undergone ablation embolization, 18 of 348 (5.2%) patients had residual tumor, and 98 of 272 (36.0%) had new tumor events.

### E2F2 had a high expression in HCC

**Fig. 1:**
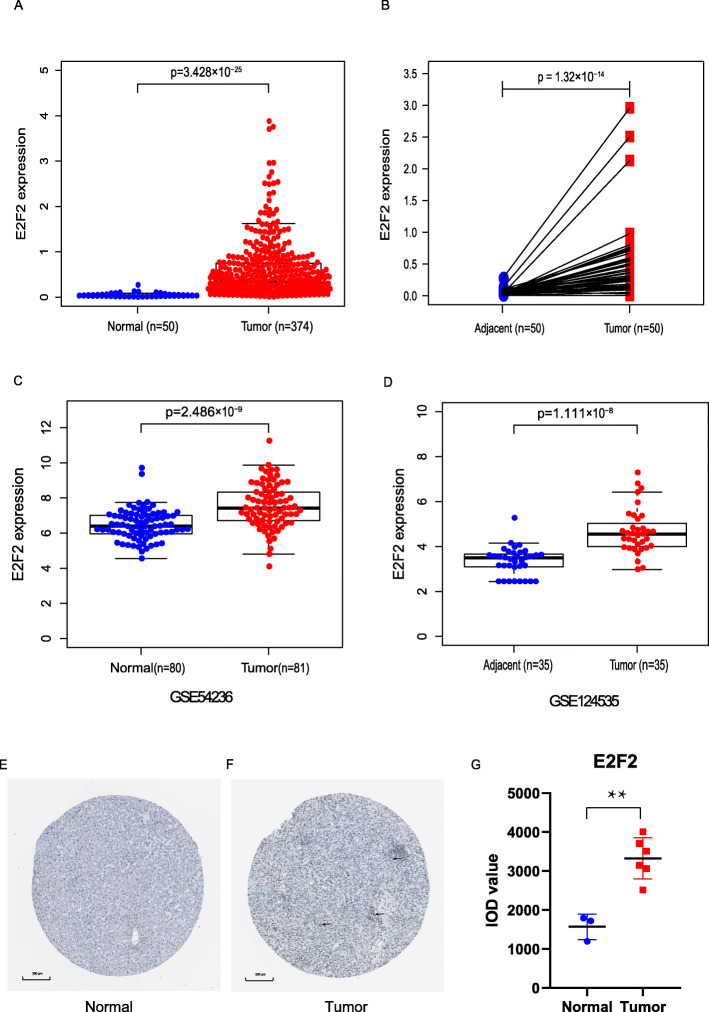
E2F2 had a high expression in HCC. **a** E2F2 showed prominently high expression in HCC tissues than in normal tissues (*p* = 3.428e−25) based on Wilcoxon rank sum test. **b** The expression of E2F2 was significantly increased in HCC tissues compared with adjacent non-cancerous tissues based on Wilcoxon signed-rank test. **c** and **d** showed E2F2 was prominently overexpressed in HCC samples from GSE124535 and GSE54236. **e** and **f** validation of protein expression levels of hub genes in the HPA database. Original magnification was × 200 μm.((E) https://www.proteinatlas.org/ENSG00000007968-E2F2/tissue/liver#img; (F) https://www.proteinatlas.org/ENSG00000007968-E2F2/pathology/liver+cancer#img); **g** IOD level of hub genes in IHC sample images. ^**^p < 0.01 compared with normal. IHC: immunohistochemistry; IOD: integrated optical density; T = topography distribution; N = lymph node metastasis; M = distant metastasis; AFP = alpha fetal protein

In the present study, we applied the Wilcoxon rank sum test to compare the expression of E2F2 in 374 HCC tissues and 50 normal tissues. As shown in Fig. 1a, the expression of E2F2 was prominently upregulated in HCC (*p* = 3.428e− 25). Besides, we investigated E2F2 expression in 50 HCC tissues and 50 adjacent normal tissues via Wilcoxon signed-rank tests, E2F2 showed significantly higher expression in HCC tissues (*p* = 1.32e− 14) (Fig. 1b). Further, in order to validate the above results, we downloaded RNA-seq and microRNA raw data from GEO database respectively, namely GSE124535 and GSE54236. The results also showed that E2F2 was prominently overexpressed in HCC samples (Fig. 1c-d).

The protein expression level of E2F2 was analyzed using IHC samples from the HPA online database. The results of HPA showed that E2F2 was mainly expressed in the nucleus. The protein level of E2F2 was upregulated in HCC tissues in comparison with normal tissues (Fig. 1 e-g), indicating that the mRNA and protein expression of E2F2 were similar in different database.

### E2F2-associated PPI network

**Fig. 2:**
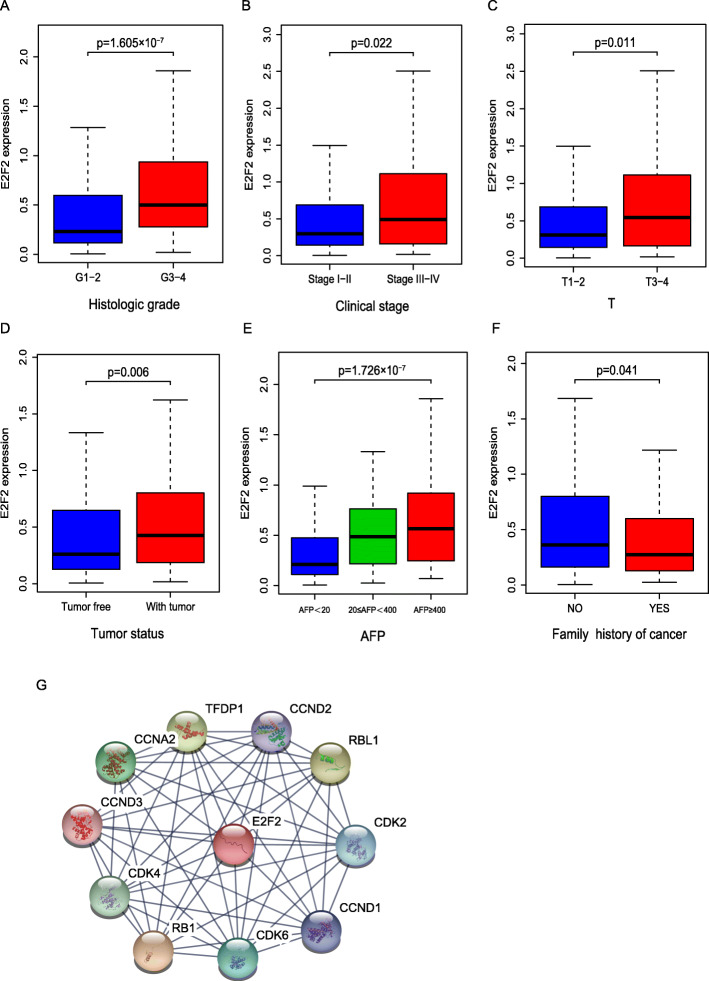
Association between E2F2 expression and clinicopathologic characteristics. As we can see from Fig. 2 (**a**–**f**), high level of E2F2 was significantly correlated with **a** histologic grade, **b** clinical stage, **c** topography, **d** tumor status, **e** AFP, **f** family history. **g** E2F2-associated PPI network. T = topography distribution; N = lymph node metastasis; M = distant metastasis; AFP = alpha fetal protein; PPI = Protein-protein interaction

An E2F2-associated PPI network was constructed based on the STRING database. As it shown in Fig. 2g, several genes had a close association with E2F2, such as CDK2, CDK4, CDK6, RB1, RBL1, CCNA2 and so on.

### The effects of overexpressed E2F2 on clinicopathological characteristics

As we can see from Fig. 2 a–f, high level of E2F2 was significantly correlated with histologic grade (*p* = 1.605e− 07), clinical stage (*p* = 0.022), T (T1/T2 vs. T3/T4, *p* = 0.011), tumor status (*p* = 0.006), AFP (*p* = 1.726e− 07), family history (*p* = 0.041).

The expression of elevated E2F2 in HCC samples was prominently correlated with histologic grade (OR = 2.62 for G3–4 vs. G1–2, *p* = 1.80E-05), clinical stage (OR = 1.74 for III-IV vs. I-II, *p* = 0.03), T (OR = 1.64 for T3–4 vs.T1–2, p = 0.04), tumor status (OR = 1.88 for with tumor vs. tumor free, *p* = 3.79E-03), AFP (OR = 3.18 for AFP ≥ 400 vs AFP<20, *p* = 2.16E-04; OR = 2.50 for 20 ≤ AFP<400 vs AFP<20, *p* = 2.56E-03). Univariate analysis using logistic regression uncovered that increased E2F2 expression (based on median expression value) was correlated with poor prognostic clinicopathologic characteristics (Table 2). This uncovered that the HCC patients with high E2F2 are tend to progress to a more advanced stage.

**Table 2:** Association between E2F2 expression and clinicopathologic characteristics (logistic regression)

Clinical characteristics	Total(N)	Odds ratio in E2F2 expression	*p*-Value
Age (>40 vs. ≤40)	370	1.48 (0.73–3.09)	0.28
Gender (male vs. female)	371	0.72 (0.46–1.11)	0.14
BMI(≥25 vs.<25)	335	0.96 (0.63–1.48)	0.87
Family history of cancer (yes vs. no)	320	0.85 (0.53–1.34)	0.48
Histologic grade (G3–4 vs. G1–2)	366	2.62 (1.69–4.10)	1.80E-05
Clinical stage (III-IV vs. I-II)	347	1.74 (1.07–2.85)	0.03
T (T3–4 vs. T1–2)	368	1.64 (1.02–2.65)	0.04
N (N1 vs. N0)	256	1.00 (0.12–8.44)	1.00
M (M1 vs. M0)	270	0.33 (0.02–2.60)	0.34
Residual tumor (R1–2 vs. R0)	342	2.08 (0.79–6.08)	0.15
Tumor status (with tumor vs. tumor free)	352	1.88 (1.20–2.89)	3.79E-03
Vascular invasion (yes vs. no)	315	1.23 (0.77–1.96)	0.38
Child-Pugh (B-C vs. A)	239	1.01 (0.42–2.45)	0.98
AFP			
AFP ≥ 400 vs. AFP<20	217	3.18 (1.74–5.94)	2.16E-04
20 ≤ AFP < 400 vs. AFP<20	219	2.50 (1.39–4.58)	2.56E-03
AFP ≥ 400 vs. 20 ≤ AFP < 400	132	1.27 (0.62–2.61)	0.51
New tumor event (yes vs. no)	269	1.48 (0.97–2.26)	0.07
Tumor weight			
W > 1000 vs. W ≤ 500	268	1.26 (0.51–3.23)	0.62
1000 ≥ W > 500 vs. W ≤ 500	278	1.18 (0.55–2.55)	0.67
W > 1000 vs.1000 ≥ W > 500	50	1.07 (0.34–3.38)	0.91
Virus			
HBV&HCV vs. HBV	141	1.13 (0.58–2.21)	0.72
HCV vs. HBV	79	0.62 (0.21–1.68)	0.35
HBV&HCV vs. HCV	104	1.83 (0.70–5.07)	0.23
History of alcohol consumption (yes vs. no)	358	1.08 (0.69–1.68)	0.73
Postoperative ablation embolization (yes vs. no)	353	1.59 (0.73–3.61)	0.25
Radiation therapy (yes vs. no)	352	1.69 (0.41–8.33)	0.48

T = topography distribution; N = lymph node metastasis; M = distant metastasis; *AFP* Alpha fetal protein

### Survival outcomes

#### Survival outcomes based on Kaplan-Meier survival analysis

**Fig. 3:**
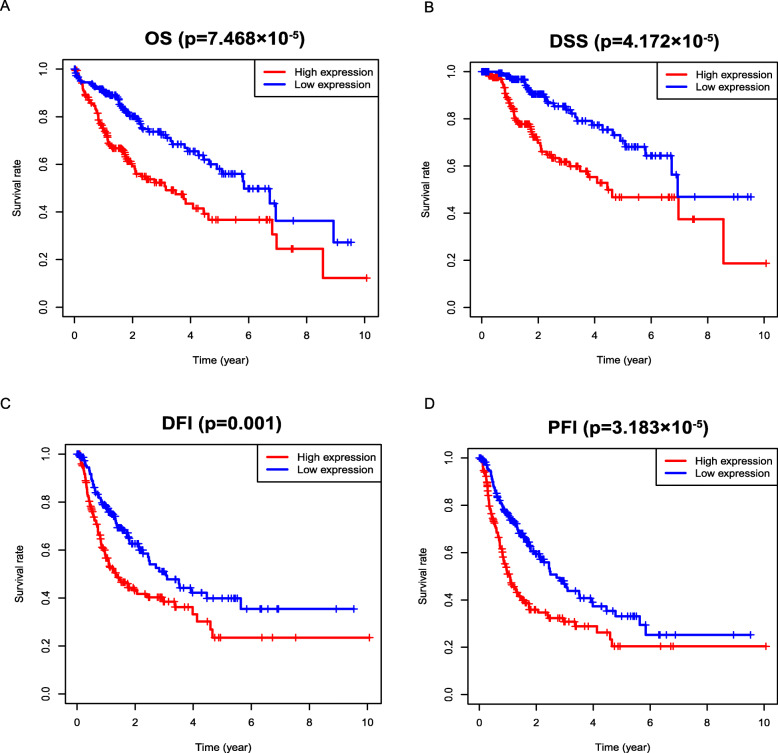
Survival outcomes based on Kaplan-Meier survival analysis. Kaplan-Meier survival analysis showed that increased E2F2 was significantly associated with poor **a** OS, **b** DSS, **c** DFI, **d** PFI. OS = overall survival; DSS = disease-specific survival; DFI = disease-free interval; PFI = progression-free interval

Kaplan-Meier survival analysis showed that increased E2F2 was significantly associated with poor OS (*p* = 7.468e− 05), PFI (*p* = 3.183e− 05), DFI (*p* = 0.001), DSS (*p* = 4.172e− 05), which indicated that HCC patients with high-E2F2 may have a worse prognosis than that with low-E2F2 (Fig. 3a-d).

#### OS, DFI and PFI outcomes using univariate and multivariate analysis with the cox regression survival model

Univariate and multivariate analysis with the Cox regression model was employed to uncover the association between clinicopathologic characteristics and HCC patient survival.

**Fig. 4:**
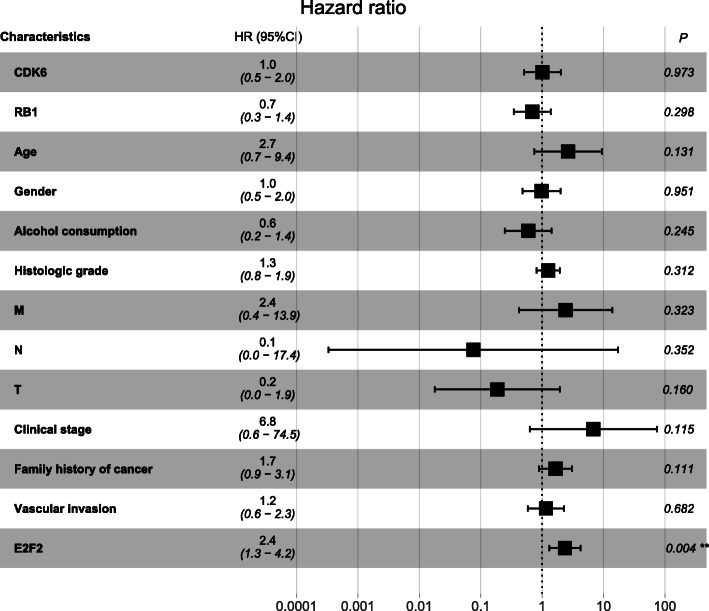
Association between clinicopathologic characteristics and HCC patient survival outcome through multivariate analysis with Cox regression survival model. It showed that worse OS was only significantly associated with high expression of E2F2 in multivariate analysis. **p < 0.01. OS = overall survival

At univariate Cox analysis, shorter overall survival (OS) was found in high expression of E2F2 (*P* = 0.002, HR = 2.0 (95% CI [1.3–3.2])), poorer TNM (T: *P* = 0.033, HR =1.4 (95% CI [1.0–2.0])), worse clinical stage (*P* = 0.012, HR = 1.5 (95% CI [1.1–2.1])) (Table 3). However, worse OS was only significantly associated with high expression of E2F2 in multivariate analysis, with a HR of 2.4 (*P* = 0.004, 95% CI [1.3–4.2]) (Table 3, Fig. 4).

**Table 3:** Association between clinicopathologic characteristics and HCC patient OS through univariate and multivariate analysis with Cox regression survival model

Characteristics	Univariate analysis			Multivariate analysis		
	HR	95%CI	*P*-value	HR	95%CI	*P*-value
CDK6 (high vs. low)	0.8	0.4–1.4	0.400	1.0	0.5–2.0	0.973
RB1 expression (high vs. low)	0.7	0.4–1.3	0.230	0.7	0.3–1.4	0.298
Age (>40 vs. ≤ 40)	2.1	0.6–6.8	0.224	2.7	0.7–9.4	0.131
Gender (male vs. female)	0.6	0.3–1.1	0.082	1.0	0.5–2.0	0.951
Alcohol consumption (yes vs. no)	0.7	0.3–1.6	0.381	0.6	0.2–1.4	0.245
Histologic grade (G3–4 vs. G1–2)	1.3	0.8–1.9	0.293	1.3	0.8–1.9	0.312
M (M1 vs. M0)	4.8	0.7–35.5	0.122	2.4	0.4–13.9	0.323
N (N1 vs. N0)	3.8	0.5–28.0	0.188	0.1	0.0–17.4	0.352
T (T3–4 vs. T1–2)	1.4	1.0–2.0	0.033	0.2	0.0–1.9	0.160
Clinical stage (III-IV vs. I-II)	1.5	1.1–2.1	0.012	6.8	0.6–74.5	0.115
Postoperative ablation embolization (yes vs. no)	1.1	0.4–2.9	0.800			
Radiation therapy (yes vs. no)	1.2	0.2–9.0	0.997			
Family history of cancer (yes vs. no)	1.5	0.8–2.8	0.197	1.7	0.9–3.1	0.111
Vascular invasion (yes vs. no)	1.3	0.7–2.5	0.384	1.2	0.6–2.3	0.682
E2F2 (high vs. low)	2.0	1.3–3.2	0.002	2.4	1.3–4.2	0.004

*OS* Overall survival, T = topography distribution, N = lymph node metastasis; M = distant metastasis, *CI* Confidence interval

**Fig. 5:**
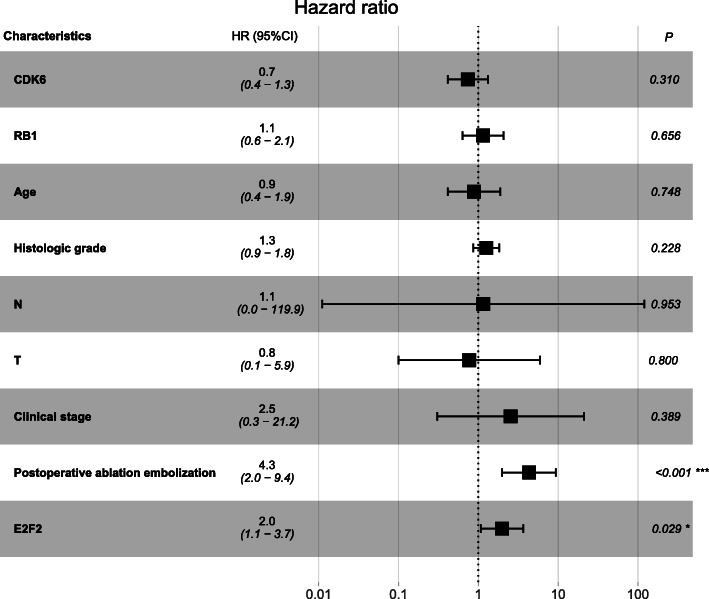
Association between clinicopathologic characteristics and HCC patient survival outcome through multivariate analysis with Cox regression survival model. At multivariate Cox analysis, poorer DFI were significantly correlated with highly expressed E2F2 and postoperative ablation embolization. *p < 0.05 and ***p < 0.001. DFI = disease-free interval

At univariate Cox analysis, poorer disease-free interval (DFI) was prominently correlated with high expression of E2F2 (*P* = 0.004, hazard ratio [HR] = 2.2 (95% CI [1.3–3.7])), higher TNM (T: *P* = 0.001, HR =1.6 (95% CI [1.2–2.1])), advanced clinical stage(*P* = 0.000, HR = 1.7 (95% CI [1.3–2.3])) and postoperative ablation embolization (*P* = 0.001, HR = 3.1 (95% CI [1.6–6.0])) (Table 4). At multivariate analysis, E2F2 (*P* = 0.029, hazard ratio [HR] = 2.0 (95% CI [1.1–3.7])) and postoperative ablation embolization (*P* = 0.000, hazard ratio [HR] = 4.3 (95% CI [2.0–9.4])) were the clinicopathologic characteristics that remained significantly correlated with DFI (Table 4, Fig. 5).

**Table 4:** Association between clinicopathologic characteristics and HCC patient DFI through univariate and multivariate analysis with Cox regression survival model

Characteristics	Univariate analysis			Multivariate analysis		
	HR	95%CI	*P*-value	HR	95%CI	*P*-value
CDK6 (high vs. low)	0.7	0.4–1.1	0.147	0.7	0.4–1.3	0.310
RB1 expression (high vs. low)	0.7	0.4–1.2	0.213	1.1	0.6–2.1	0.656
Age (>40 vs. ≤ 40)	0.6	0.3–1.2	0.167	0.9	0.4–1.9	0.748
Gender (male vs. female)	1.0	0.6–1.6	0.862			
Alcohol consumption (yes vs. no)	1.0	0.6–1.9	0.915			
Histologic grade (G3–4 vs. G1–2)	1.4	1.0–1.9	0.083	1.3	0.9–1.8	0.228
M (M1 vs. M0)	5.5	0.7–40.4	0.722			
N (N1 vs. N0)	3.9	0.5–28.6	0.180	1.1	0.0–119.9	0.953
T (T3–4 vs. T1–2)	1.6	1.2–2.1	0.001	0.8	0.1–5.9	0.800
Clinical stage (III-IV vs. I-II)	1.7	1.3–2.3	0.000	2.5	0.3–21.2	0.389
Postoperative ablation embolization (yes vs. no)	3.1	1.6–6.0	0.001	4.3	2.0–9.4	0.000
Radiation therapy (yes vs. no)	1.5	0.2–10.9	0.690			
Vascular invasion (yes vs. no)	1.2	0.7–2.1	0.524			
Family history of cancer (yes vs. no)	1.2	0.7–2.0	0.513			
E2F2 (high vs. low)	2.2	1.3–3.7	0.004	2.0	1.1–3.7	0.029

*DFI* Disease-free interval, T = topography distribution, N = lymph node metastasis, M = distant metastasis, *CI* Confidence interval

**Fig. 6:**
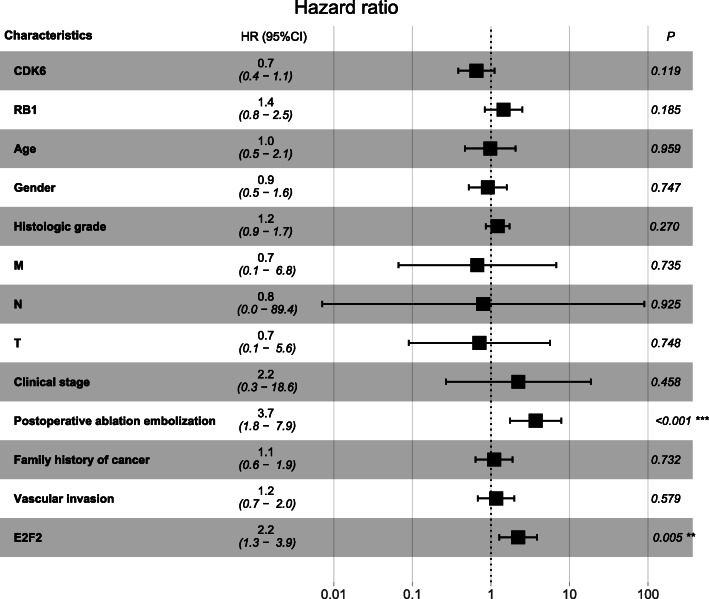
Association between clinicopathologic characteristics and HCC patient survival outcome through multivariate analysis with Cox regression survival model. The multivariate Cox analysis showed that shorter PFI were significantly associated with high expression of E2F2 and postoperative ablation embolization. ***p < 0.001. PFI = progression-free interval

The univariate Cox analysis indicated that highly expressed E2F2 was significantly associated with worse PFI (*P* = 0.005, hazard ratio [HR] = 1.8 (95% CI [1.2–2.6])). Other clinicopathological characteristics such as higher TNM (T: *P* = 0.001, HR =1.5 (95% CI [1.2–2.0])), advanced clinical stage(*P* = 0.000, HR = 1.6 (95% CI [1.2–2.1])) and postoperative ablation embolization (*P* = 0.001, HR = 2.8 (95% CI [1.5–5.2])) were also associated with poor survival (Table 5). The multivariate Cox analysis showed that E2F2 (*P* = 0.005, hazard ratio [HR] = 2.2 (95% CI [1.3–3.9])) and postoperative ablation embolization (*P* = 0.001, hazard ratio [HR] = 3.7 (95% CI [1.8–7.9])) were the clinicopathologic characteristics that remained significantly correlated with PFI (Table 5, Fig. 6).

**Table 5:** Association between clinicopathologic characteristics and HCC patient PFI through univariate and multivariate analysis with Cox regression survival model

Characteristics	Univariate analysis			Multivariate analysis		
	HR	95%CI	*P*-value	HR	95%CI	*P*-value
CDK6 (high vs. low)	0.7	0.4–1.1	0.082	0.7	0.4–1.1	0.119
RB1 expression (high vs. low)	0.8	0.5–1.3	0.374	1.4	0.8–2.5	0.185
Age (>40 vs. ≤ 40)	0.7	0.4–1.3	0.268	1.0	0.5–2.1	0.959
Gender (male vs. female)	0.7	0.5–1.2	0.239	0.9	0.5–1.6	0.747
Alcohol consumption (yes vs. no)	0.9	0.5–1.6	0.804			
Histologic grade (G3–4 vs. G1–2)	1.4	1.0–1.9	0.061	1.2	0.9–1.7	0.270
M (M1 vs. M0)	4.9	0.7–36.2	0.118	0.7	0.1–6.8	0.735
N (N1 vs. N0)	3.4	0.5–24.6	0.230	0.8	0.0–89.4	0.925
T (T3–4 vs. T1–2)	1.5	1.2–2.0	0.001	0.7	0.1–5.6	0.748
Clinical stage (III-IV vs. I-II)	1.6	1.2–2.1	0.000	2.2	0.3–18.6	0.458
Postoperative ablation embolization (yes vs. no)	2.8	1.5–5.2	0.001	3.7	1.8–7.9	0.001
Radiation therapy (yes vs. no)	1.3	0.2–9.3	0.806			
Family history of cancer (yes vs. no)	1.2	0.7–2.0	0.445	1.1	0.6–1.9	0.732
Vascular invasion (yes vs. no)	1.4	0.8–2.2	0.228	1.2	0.7–2.0	0.579
E2F2 (high vs. low)	1.8	1.2–2.6	0.005	2.2	1.3–3.9	0.005

*PFI* Progression-free interval, T = topography distribution, N = lymph node metastasis, M = distant metastasis, *CI* Confidence interval

### Identification of E2F2 related signaling pathway by GSEA

Gene Set Enrichment Analysis (GSEA) was applied to extract prominently activated signaling pathways between low and high E2F2 expression data sets, and validated their significant differences (FDR < 0.05, NOM *P*-value < 0.05) in enrichment of MSigDB Collection (c2.cp.v6.2.symbols.gmt). 10 signaling pathways with significant differences, including the cell circle, the RNA degradation, the pyrimidine metabolism, the base excision repair, the aminoacyl tRNA biosynthesis, the DNA replication, the p53 signaling pathway, the nucleotide excision repair, the ubiquitin mediated proteolysis, the citrate cycle TCA cycle were filtered out, which were prominently enriched in E2F2 high expression phenotype based on NES, NOM P-value, and FDR value (Fig. 4a–b, Fig. 5; Table 5).

## Discussion

The root cause of cancer is the accumulation of genetic mutations [16], it is speculated that more than two-thirds of the mutations in tumor tissue result from DNA replication errors during cell proliferation [17]. Uncontrolled proliferation, apoptotic disorder, immortalized replication, long-lasting angiogenesis, local invasion, distant metastasis, escape from the immune and growth inhibitors, and so on are the biological capabilities that cancer acquires during the development process. The basis of these capabilities is genetic instability and chronic inflammation [18]. The E2F family is downstream of CDK-E2Fs-Rb network in a cell cycle regulation network [19, 20], and is a crucial transcriptional regulatory factor in the cell cycle. It has been reported that they not only play an important role in cell proliferation and maintain gene stability [17], but also have critical effects on apoptosis, metabolism, differentiation, DNA damage and repair, angiogenesis and so on [21, 22]. The role of the E2F family is very complex, they seem to act as tumor suppressors or promoters depending on their environment, target genes and coenzyme factors [23].

As an important member of the E2F family, E2F2 is considered to be a transcriptional activator of the target gene of E2F. It regulates the transcriptional activity of the target gene by binding to the promoter of the target gene, and plays a key role in regulating G1 / S phase transition and the beginning of DNA replication [24]. Previous studies have shown that E2F2 is an oncogene in many tumor types, for instance, it has been discovered that E2F2 is prominently up-regulated in NSCLC, and can be serve as a therapeutic target to prevent the proliferation and invasion of NSCLC [4]. Quan Zhou et al. reported that overexpressed E2F2 is closely related to poor post progression survival in ovarian cancer patients, and can be used for targeted treatment and prognosis prediction [5]. Additionally, Hang Song et al. provided evidence that Let-7b can inhibit the malignant proliferation of glioma cells by down-regulating the expression of E2F2 [6]. Similar results can be found in osteosarcoma [7], gastric cancer [8] and melanoma [9].

However, so far, there has been little research on the role of E2F2 in HCC. An experimental study reported that mir-218 and mir-520a could inhibit the proliferation of HCC cells by down-regulating the expression of E2F2 [25], it implied that highly expressed E2F2 is associated with the proliferation of HCC. Another study provided evidence that overexpression of mir-490-5p inhibited the metastasis of HCC cells by down-regulating the expression of E2F2 and ECT2 [26], this study indirectly suggests that E2F2 may be involved in the metastasis of HCC cells. Nevertheless, none of them systematically investigated the role of E2F2 in HCC. Seong Hwi Hong et al. [27] concluded that E2F2 was highly expressed in HCC based on the data analysis of GEO database, and suggested that high E2F2 expression was associated with poor OS by Kaplan-Meier plot. Unfortunately, this study has its limitations. It did not analyze the correlation between E2F2 and other clinicopathological characteristics of HCC patients. In terms of survival analysis, E2F2 was only proposed to be associated with poor OS, other survival outcomes like DFI, PFI and DSS were not considered. Besides, most importantly, they did not use multivariate regression analysis. There have been no other reports on the value of E2F2 in predicting the prognosis of HCC. The underlying mechanism by which E2F2 is closely associated with HCC has not been elucidated completely. Our study investigated the expression of E2F2 in HCC based on TCGA database, we found that E2F2 is overexpressed at both the mRNA and protein levels (Fig. 1e-g). Subsequently, we further analyzed the relationship between E2F2 expression and the clinicopathological characteristics of HCC patients, and the effect of high E2F2 expression on the prognosis of HCC patients. Our study revealed that high E2F2 expression was closely related to the worse histologic grade, advanced clinical stage, more lymph node metastasis, and higher serum AFP value (Fig. 2, Table 2). Moreover, our study uncovered that elevated E2F2 was negatively correlated with OS, DFI, PFI and DSS (Fig. 3). Most importantly, multivariate regression analysis provided evidence that highly expressed E2F2 was strikingly associated with poor OS, PFI and DFI even after other factors were excluded (Figs. 4, 5 and 6, Tables 3, 4 and 5), suggesting that E2F2 can independently predict the prognosis of HCC patients.

**Fig. 7:**
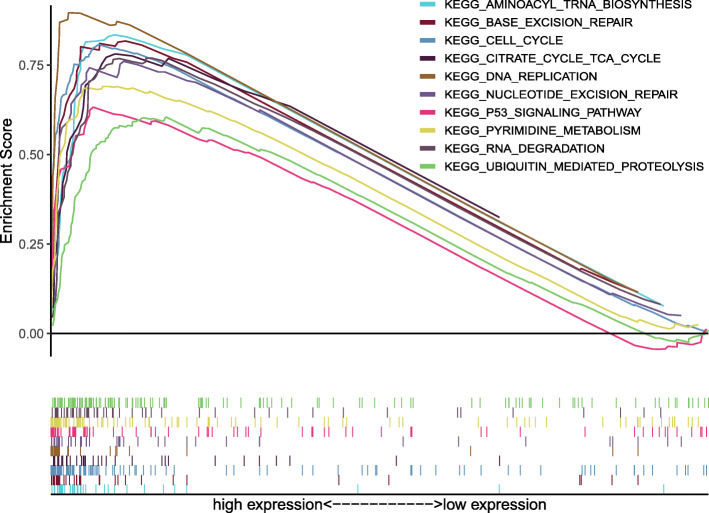
Enrichment plots from gene set enrichment analysis (GSEA). Results of GSEA showed the cell circle, the RNA degradation, the pyrimidine metabolism, the base excision repair, the aminoacyl tRNA biosynthesis, the DNA replication, the p53 signaling pathway, the nucleotide excision repair, the ubiquitin mediated proteolysis, the citrate cycle TCA cycle were differentially enriched in E2F2-related HCC. ES = enrichment score; NES = normalized ES; FDR = false discovery rate; NOM *p*-val = normalized *p*-value

In the present study, cell cycle, RNA degradation, pyrimidine metabolism, base excision repair, aminoacyl tRNA biosynthesis, DNA replication, p53 signaling pathway, nucleotide excision repair, ubiquitin mediated proteolysis and citrate cycle TCA cycle were the major pathway regulated by E2F2 based on GSEA (Fig. 7, Table 6).

**Table 6:** Gene sets enriched in phenotype high

MSigDB collection	Gene set name	NES	NOM p-val	FDR q-val
c2.cp.kegg.v7.0.symbols.gmt[Curated]	KEGG_CELL_CYCLE	2.400	0.000	0.000
	KEGG_RNA_DEGRADATION	2.274	0.000	0.001
	KEGG_PYRIMIDINE_METABOLISM	2.273	0.000	0.001
	KEGG_BASE_EXCISION_REPAIR	2.224	0.000	0.002
	KEGG_AMINOACYL_TRNA_BIOSYNTHESIS	2.163	0.000	0.004
	KEGG_DNA_REPLICATION	2.149	0.000	0.005
	KEGG_P53_SIGNALING_PATHWAY	2.116	0.000	0.005
	KEGG_NUCLEOTIDE_EXCISION_REPAIR	2.101	0.000	0.006
	KEGG_UBIQUITIN_MEDIATED_PROTEOLYSIS	2.012	0.004	0.012
	KEGG_CITRATE_CYCLE_TCA_CYCLE	1.995	0.006	0.013

*NES* Normalized enrichment score, *NOM* Nominal, *FDR* False discovery rate. Gene sets with NOM *p*-val < 0.05 and FDR q-val < 0.05 are considered as significant.

The possible role of abnormal E2F2 in the regulation of cell cycle and DNA replication in HCC have been described above. Besides, pyrimidine metabolism, p53 signaling pathway and ubiquitin-mediated protease are pathways that are also closely correlated with the regulation of cell cycle. p53 is by far one of the most important tumor suppressors. p53 and its target genes constitute a complex p53 signaling pathway that regulates various biological functions, such as DNA repair, cell cycle regulation, cell apoptosis, aging, and energy metabolism, in order to maintain gene integrity and prevent tumor formation. Almost all types of tumors and more than 50% of human tumor cells have p53 mutations. The p53 mutation and subsequent regulation of its target genes cause the p53 signaling pathway not only lose the effect of tumor inhibition, but also acquire carcinogenic functions, such as promoting cell proliferation, metastasis, anti-apoptosis, angiogenesis and metabolic changes [28]. Literature has been reported that genes in the p53 signaling pathway and cell cycle signaling pathway are often mutated in HCC [29]. In other words, the p53 signaling pathway and cell cycle signaling pathway are often dysregulated in HCC. Dysregulation of these signaling pathways is frequently involved in the development and progression of HCC. In addition, E2F2 has been previously reported to have regulatory effects on p53. Abnormal DNA replication in E2F1/2 knockout cells can activate the p53 pathway and then generate p53-dependent apoptosis to prevent the occurrence of tumor, but when p53 is also inactivated, it promotes tumor development. The powerful E2F-p53 regulatory axis has the function of maintaining tissue homeostasis and preventing tumorigenesis [30]. Another reports suggested that the targeted inactivation of E2F1–3 leads to cell cycle stagnation at G1 / S and G2 / M, and when p53 and p21 are also inactivated, cells resume cell cycle progression and continue to grow. The inactivation of E2F1–3 activates the p53-p21 axis, they together control the process of the cell cycle and prevents the occurrence of tumors [31]. Besides, as a component of many key molecules, pyrimidines are involved in important biological processes such as the synthesis of DNA, RNA, saccharides and lipid [32]. Abnormal pyrimidine pathways can promote the characteristics of cancer stem cells in poorly differentiated HCC, which can be used as a potential therapeutic target for anti-HCC tumor progression [33]. Additionally, ubiquitin-mediated proteolysis has the function of regulating and controlling the normal evolution of cell cycle, and the maladjustment of this pathway can lead to abnormal cell proliferation, gene instability and the occurrence of cancer [19]. Studies have revealed that genes in this signaling pathway, such as HUWE1, are often mutated in HCC and are associated with the proliferation of HCC [34]. Previous studies have explored the regulation of E2F2 expression on cell cycle, DNA replication and p53 signaling pathway, nevertheless, at present, no literature has revealed the relationship between E2F2 expression and pyrimidine metabolism, ubiquitin mediated protease. Our study is the first to report the regulatory effects of E2F2 on pyrimidine metabolism, ubiquitin mediated protease and p53 signaling pathway in HCC, and this regulatory mechanism needs to be further verified by experiments.

TCA cycle, also known as the citric acid cycle or Krebs cycle, is an important pathway for substance metabolism and energy supply in the human body. About two-thirds of the organic substances in the human body, including three major nutrients (sugar, fat and protein) are decomposed by TCA cycle. It is also a common pathway for the complete oxidation and decomposition of the three major nutrients to provide energy. Early studies suggested that cancer cells bypass the TCA cycle and use aerobic glycolysis, but emerging evidence suggests that some cancer cells, particularly those with the maladjusted expression of oncogenes and tumor suppressors, rely heavily on the TCA cycle to produce energy and synthesize large molecules [35]. In a variety of cancers, including HCC, the expression or activity levels of the TCA cycle and related enzymes are generally dysregulated, which is a pivotal driver of cancer development and progression [36, 37]. In addition, wild-type P53 also has an important effect on metabolism, the mutation of P53 will lead to the enhancement of glycolysis and the reduction of oxidative phosphorylation in tumor cells. As a result, tumor cells digest a large amount of glucose but cannot produce energy efficiently. Our study is the first to report the relationship between E2F2 and TCA cycle in HCC and it needs more work to be verified in the future.

Although our current study has improved our understanding of the role of E2F2 in HCC, there are still some limitations. Firstly, this study is a retrospective study, and prospective studies should be conducted in the future to make up for the limitations of the retrospective study. Afterwards, the absence of clinical factors in the public database, such as specific details of the patient’s medication and/or surgical treatment, also affects the patient’s prognosis. Finally, the protein level of E2F2 in HCC and its direct role in HCC progression and metastasis remain to be further validated in vitro. Although this study has some limitations, it does provide clues for studying the function of E2F2 in HCC, and provides targets and potential prognostic markers for the treatment of HCC.

## Conclusion

In our study, we systematically explored the expression of E2F2 in HCC, and confirmed that elevated E2F2 was bound up with an advanced histologic grade, clinical stage, more lymph node metastasis, higher serum AFP level and poor survival outcome (OS, DSS, DFI and PFI). Additionally, cell cycle, pyrimidine metabolism, DNA replication, p53 signaling pathway, ubiquitin mediated proteolysis, the citrate cycle TCA cycle may be the key pathway by which E2F2 participates in the initial and progression of HCC. Our findings partly disclosed the clinical significance of E2F2 in HCC and suggested that E2F2 may be a promising independent prognostic biomarker and therapeutic target for HCC. However, further experiments are needed to verify the results.

## Data Availability

The datasets generated and/or analyzed during the current study are available in the TCGA repository, https://portal.gdc.cancer.gov/repository?facetTab=cases; and GEO repository https://www.ncbi.nlm.nih.gov/geo/query/acc.cgi?acc=GSE124535 and https://www.ncbi.nlm.nih.gov/geo/query/acc.cgi?acc=GSE54236.

## Abbreviations

E2F2: E2F family of transcription factor 2
GEO: Gene Expression Omnibus
GSEA: Gene Set Enrichment Analysis
OS: Overall survival
DSS: Disease-specific survival
PFI: Progression-free interval
DFI: Disease-free interval
AFP: Alpha fetoprotein
BMI: Body Mass Index
